# Activation of the stress-activated protein kinase JNK in response to herpes simplex virus-1 infection coordinates transition of BRD4 from chromosome association to transcription elongation

**DOI:** 10.1016/j.jbc.2025.110590

**Published:** 2025-08-12

**Authors:** Weikang Sun, Ke Ren, Jingjing Li, Mengyu Zhang, Qishen Jiang, Lingling Wang, Erguang Li

**Affiliations:** 1 State Key Laboratory of Pharmaceutical Biotechnology and Medical School, Nanjing University, Nanjing, Jiangsu, China; 2 Jiangsu Key Laboratory of Molecular Medicine, Medical School, Nanjing University, Nanjing, Jiangsu, China; 3 School of Laboratory Medicine, Chengdu Medical College, Chengdu, China; 4 College of Life Sciences, Nanjing University, Nanjing, China; 5 School of Medical Informatics, Xihua University, Chengdu, Sichuan, China; 6 Institute of Medical Virology, The Affiliated Drum Tower Hospital of Nanjing University Medical School, Nanjing, China

**Keywords:** bromodomain-containing protein 4, BRD4 release, HSV-1, mitogen-activated protein kinase, c-Jun N-terminal kinase, P-TEFb

## Abstract

The c-Jun N-terminal kinase (JNK) signaling pathway is required for herpes simplex virus 1 lytic infection and reactivation from latency. JNK signaling regulates cellular processes in response to stress through transcriptional regulation. However, the mechanisms by which JNK regulates HSV-1 infection are less defined. We show here that HSV-1 infection triggers BRD4 transition from chromosome association to transcriptional regulation for viral infection. Specifically, HSV-1 infection induces JNK activation which mediates redistribution of BRD4, an epigenetic reader protein, from chromatin-targeting to association with proteins of transcriptional regulation. BRD4 transitions to viral infection regulation by complexing with P-TEFb, a positive transcription elongation factor, and association with viral DNA. Genetic ablation or perturbation of JNK with chemical inhibitors or siRNA leads to impediment of BRD4 release and inhibition of HSV-1 infection. Both chemotherapeutic agents and irradiation are known to promote JNK activation and HSV reactivation. We show further that JNK agonist or chemotherapeutic agents known to activate JNK can enhance HSV-1 infection. Our study reveals a novel mechanism by which JNK regulates HSV-1 infection through stress-induced BRD4 function transition from host chromosome association to viral gene expression. The work links recurrent HSV infection by chemotherapeutic agents to JNK activation.

Herpes simplex virus-1 and -2 (HSV-1 and HSV-2) are two members of the human Herpesviridae family. Both HSV-1 and HSV-2 are important pathogens of human diseases. HSV-1 infection is the main cause of herpes infections on the mouth and lips, including cold sores and blisters. Genital herpes is mainly caused by HSV-2, but HSV-1 infection has been up lately (1). According to reports from the World Health Organization, two thirds of world population under 50 years of age had HSV-1 infection, while over 550 million individuals of 15 to 49 years old had genital herpes by HSV-2 and HSV-1 infection (2, 3, 4).

HSV-1 infection is initiated by cell attachment followed by cell entry. Following penetration into a host cell, the viral DNA enters the nucleus and is transcribed into mRNA by cellular RNA polymerase II (Pol II) (5). During productive infection by HSV-1, more than 80 genes encoded within the linear 152-kbp viral genome are transcribed in sequential phases termed immediate early (IE; α), early (E; β), and late genes (L; γ) in a temporal cascade (6, 7). Viruses have co-evolved with their human hosts and depend on cellular, in addition to viral, proteins for replication. In particular, the mitogen-activated protein kinase (MAPK) family proteins play distinct roles in viral infection and in diseases (8, 9) since they relay, amplify and integrate signals to elicit an appropriate physiological response in complex cellular programs like proliferation, differentiation, inflammatory responses, and apoptosis (10).

At least three MAPK families have been characterized in mammalian cells, the extracellular signal-regulated kinase (ERK), c-Jun N-terminal kinase (JNK), and p38/MAPK (11). The c-Jun N-terminal kinases, also known as stress activated protein kinases (SAPKs), orchestrate complex cellular responses to stress signals, thereby influencing cell fate decisions. JNK pathway signaling serves as responders to many environmental cues and have been implicated in the pathogenesis of a number of viruses (12). Lytic HSV-1 infection stimulates JNK and p38 pathways, driving viral RNA production and viral replication (13, 14). JNK is also required for HSV reactivation from latency since JNK signaling leads to histone methyl/phospho switch on HSV lytic gene promoters to promote reactivation (15, 16, 17). However, little is known regarding the mechanisms by which JNK regulates HSV replication and lytic infection.

Epigenetic regulation also plays a critical role in HSV-1 lytic infection as well as in viral latency (18, 19). The DNA from mature herpes virion is not associated with histones but is rapidly loaded with heterochromatin upon entry into the cell nucleus during lytic infection. As a reader protein, BRD4 uses 2 tandem bromodomains to interact with acetylated lysine residues on histones and other proteins (20, 21, 22). BRD4 also uses its P-TEFb interaction domain (PID) to recruit the positive transcription elongation factor (P-TEFb) to promoters to regulate transcription elongation (23, 24). Thus, BRD4 transition from chromatin-targeting to transcription regulation is essential for signal-induced gene expression (25). It was reported that JNK activation by stress triggers release of BRD4 from chromatin targeting to transcriptional regulation under stress signaling (25, 26, 27). Released BRD4 directly interacts with transcriptional regulators, including Pol II, P-TEFb, and c-Myc, thereby promoting transcription of target genes involved in host responses (28). Various signaling cascades, including DNA damaging with chemotherapeutic agents and UV irradiation can trigger rapid JNK activation. JNK activation releases BRD4 from chromatin-targeting to transcription regulation including association with RNA Pol II for gene transcription and expression (28). Thus, stress signals from virus infection could induce BRD4 transition from host gene regulation to viral gene transcription and infection.

In this study, we investigated the signaling events required for HSV-1 infection and identified JNK as a critical molecule regulating BRD4 transition from chromatin-targeting to HSV-1 infection. We show that JNK agonists as well as chemotherapeutic agents that can activate JNK have the potential to promote HSV-1 infection. Here we report that JNK mediates HSV-1 lytic infection through BRD4 function transition.

## Results

### HSV-1 infection requires JNK/MAPK activation

**Figure 1:**
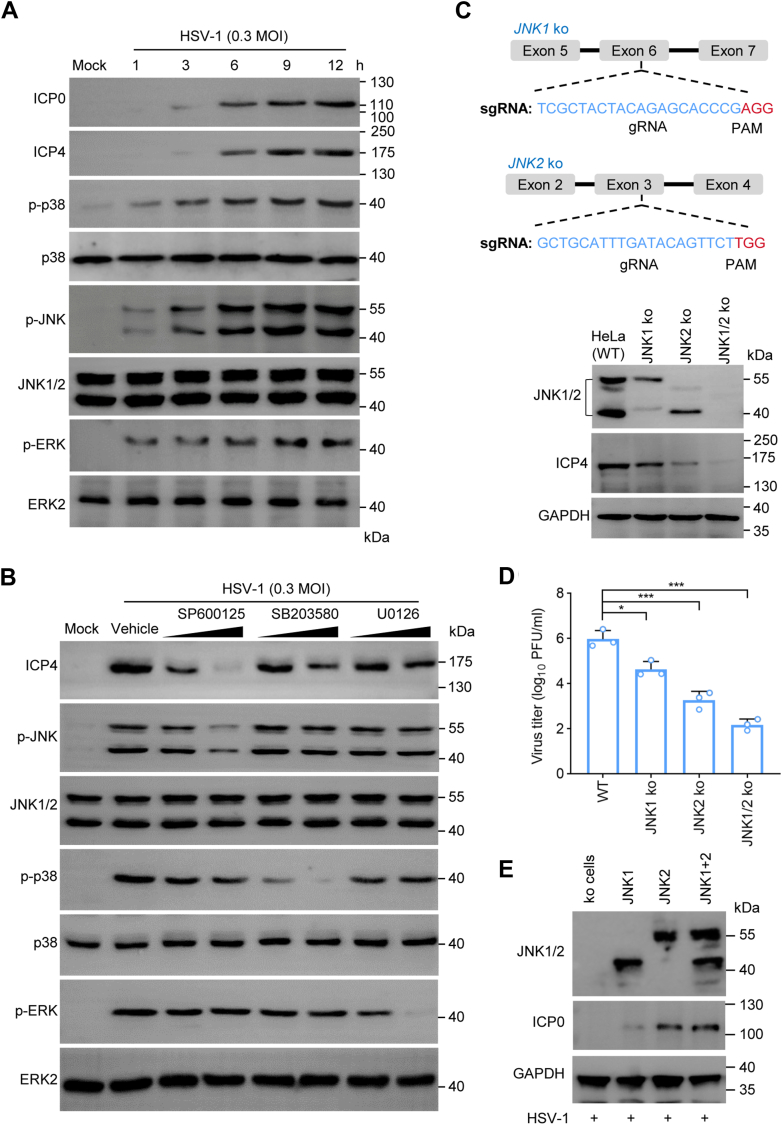
**HSV-1 infection requires JNK/MAPK pathway signaling.***A*, HSV-1 infection promotes phosphorylation of MAPK kinases. HeLa cells were infected with HSV-1 at an MOI of 0.3 for times as indicated. Protein phosphorylation and expression was detected by specific antibodies. The HSV-1 ICP0 and ICP4 were used as markers for infection. *B*, chemical inhibition of JNK on HSV-1 infection. Infected or uninfected control HeLa cells were mock-treated or treated with an MAPK inhibitor at 1 h prior to HSV-1 infection. The cells were harvested at 6 h post-infection and used for the detection of protein expression and phosphorylation. SP600125 (a JNK inhibitor, at 5 and 10 μM), SB203580 (a p38 inhibitor, at 0.3 and 1.0 μM), and U0126 (an ERK pathway inhibitor, at 1 and 3 μM). *C* and *D*, construction and confirmation of *JNK* knockout on HSV-1 infection. JNK ko HeLa cells were constructed and used for HSV-1 infection for 24 h. JNK ko and viral protein ICP4 production was determined by immunoblotting (*C*) and for infectious virus production (*D*). *E*, ectopic expression of JNK in JNK1/2 ko cells rescues HSV-1 infection. The JNK1/2 ko cells were transfected with plasmids encoding JNK1, JNK2, or both (JNK1+2) for 48 h, followed by HSV-1 infection another 36 h and then used for immunoblotting viral protein ICP0 expression. GAPDH was a loading control. The immunoblotting experiments were performed 3 times independently, while virus titers were from one representative experiment using triplicate samples. Data are mean ± SD, ∗, *p* ≤ 0.05, ∗∗∗, *p* ≤ 0.001.

JNK activity is required for HSV-1 infection and reactivation (13, 16, 29, 30). To delineate a mechanism by which JNK regulates HSV-1 lytic infection, we first performed an infection assay to confirm JNK activation at the early stages of HSV-1 infection. To this end, HeLa cells were infected with HSV-1 (MOI = 0.3) for times as indicated (Fig. 1*A*). MAPK activation was determined by assay for protein phosphorylation. We found that HSV-1 infection caused MAPK activation throughout the time points of the assay, including significant phosphorylation of JNK, p38, and to a lesser degree of ERK proteins in a time-dependent manner (Fig. 1*A*). Treatment with SP600125, a selective JNK inhibitor, and, to a lesser degree, with p38 inhibitor SB203580, blocked HSV-1 infection determined by HSV-1-infected cell protein-4 (ICP4) expression (Fig. 1*B*), indicating HSV-1 required JNK activity.

The requirement of JNK for HSV-1 infection was demonstrated using the *JNK* knockout (ko) cells. We generated *JNK1, JNK2*, and *JNK1/2* ko HeLa cell lines since JNK1 and JNK2 are ubiquitously expressed across various tissues and tested the effect in supporting HSV-1 productive infection (Fig. 1, *C* and *D*). Knockout of JNK1 or JNK2, and more profoundly, JNK1/2 expression significantly blocked HSV-1 infection as was demonstrated by reduced expression of HSV-1 ICP4 (Fig. 1*C*). The effect was confirmed by titration for infectious virus production at 24 h after HSV-1 infection. We detected more than 3-log reduction of infectious virion in JNK1/2 ko cells, while those from JNK1 and JNK2 ko cells had 1.2 and 1.7-log reduction, respectively (Fig. 1*D*). Importantly, the defect of the ko cells in supporting HSV-1 infection was partially rescued by ectopic re-expression of JNK (Fig. 1*E*). These, together with previous reports (8, 29, 31), demonstrated that HSV-1 infection required JNK activity.

### JNK activation by HSV-1 infection promotes BRD4 release

**Figure 2:**
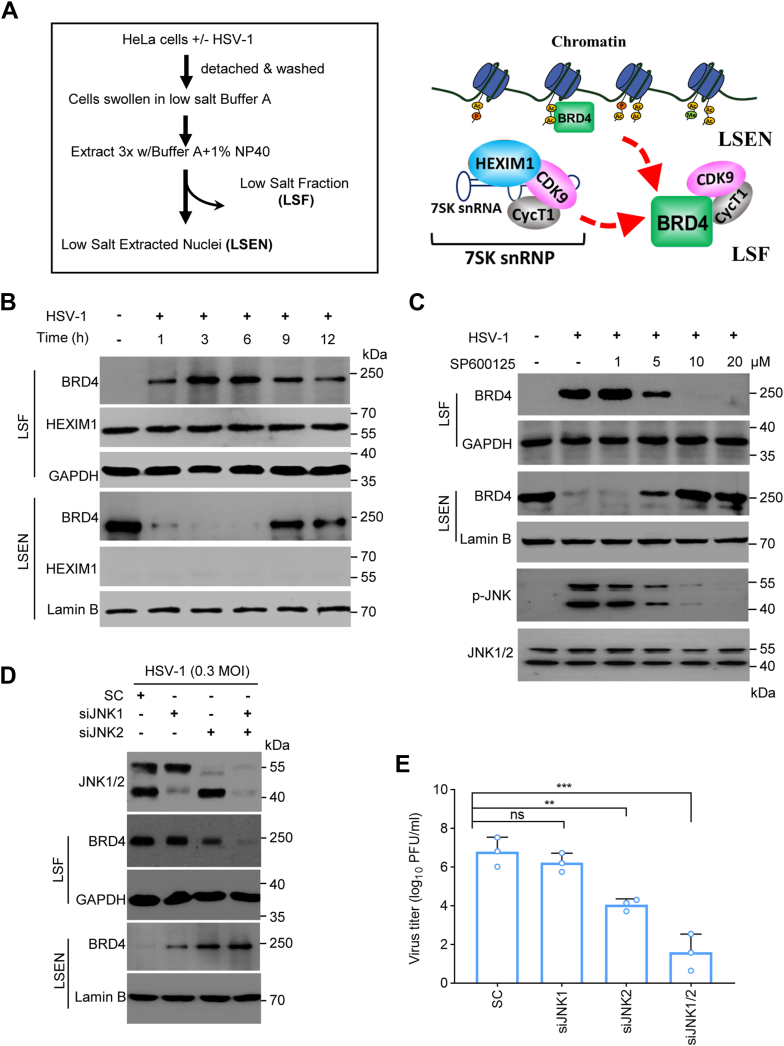
**HSV-1 infection promotes BRD4 release from chromatin-targeting dependent on JNK signaling.***A*, schematic diagram for chromatin-targeting and release of BRD4 by fractionation. *B*, HSV-1 infection releases BRD4 from chromatin-targeting. HeLa cells were infected with HSV-1 (MOI = 1) for times as indicated and used for fractionation. BRD4 in the LSF and LSEN fractions was detected by immunoblotting. GAPDH, Lamin B and HEXIM1 were used as controls for fractionation. *C*, JNK inhibition on HSV-1 induced BRD4 release. HeLa cells were infected with HSV-1 for 6 h, and were then mock-treated or treated with SP600125 for another hour prior to fractionation. BRD4 in the LSF and LSEN fractions was detected by immunoblotting. GAPDH and Lamin B were used as controls. *D* and *E*, suppression of JNK expression by siRNA on HSV-1-induced BRD4 release (*D*) and HSV-1 infection (*E*).The experiments were performed 3 times independently, The experiments were performed 3 times independently. The titration data are presented as mean ± SD from one experiment. ns, no significant, ∗∗, *p* ≤ 0.01, ∗∗∗, *p* ≤ 0.001.

A recent report demonstrated JNK phosphorylates BRD4 in response to chemical and physical stimulation to switch its function from chromatin-targeting to transcriptional activation (25, 26). BRD4 is an epigenetic reader protein that we and others have demonstrated to have an essential role in herpesvirus infection (19, 32). We asked whether JNK activation promoted BRD4 release from chromosome targeting by HSV-1 infection. In this regard, we first performed a fractionation following a reported protocol (25) to determine if HSV-1 infection promoted BRD4 release through JNK activation (Fig. 2*A*). As shown in Figure 2*B*, BRD4 was mainly detected in the low salt extracted nuclei fraction (LSEN) from the uninfected control samples, while little to no BRD4 was detected in the low salt fraction (LSF) from these samples, indicating that BRD4 was in a state of chromosome association in quiescent cells. For comparison, HSV-1 infection promoted BRD4 release since significant accumulation of BRD4 was detected in the LSF fractions from HSV-1 infected samples, indicating HSV-1 infection induced BRD4 release from chromatin targeting (Fig. 2*B*).

Next, we addressed whether JNK was required to mediate infection-induced BRD4 release since a recent report showed that JNK was required to modulate anisomycin-induced BRD4 release for transcriptional regulation (28). We thus included samples by treatment with varying amount of SP600125 to block JNK activation (Fig. 2*C*). Consistent with the observation that JNK was required for productive HSV-1 infection, we found that JNK inhibition with SP600125 dose-dependently blocked BRD4 redistribution to the LSF. The effect was further confirmed by silencing JNK expression using siRNA. As shown in Figure 2*D*, suppression of JNK1 or JNK2 expression reduced HSV-1-induced BRD4 release, while combined suppression of both JNK1 and JNK2 expression overwhelmingly blocked BRD4 redistribution. Similar to genetic ablation, JNK suppression by siRNA also blocked productive HSV-1 infection (Fig. 2*E*). Thus, these results together demonstrated that JNK was required for HSV-1 lytic infection as well as BRD4 release from chromatin-targeting.

It was of noting that anisomycin-induced BRD4 release from chromatin-targeting required BDR4 phosphorylation at T1186 and T1212 sites (28). We found transient expression of BRD4 T1186A/T1212A mutant had little effect on HSV-1 infection (Fig. S1). Although the mechanisms by which HSV-1 induced BRD4 release is regulated remain undefined, this suggested to us that HSV-1 might use different mechanisms to modulate BRD4 release.

### HSV-1 infection induces complex formation containing BRD4-P-TEFb-Pol II and association with viral DNA

**Figure 3:**
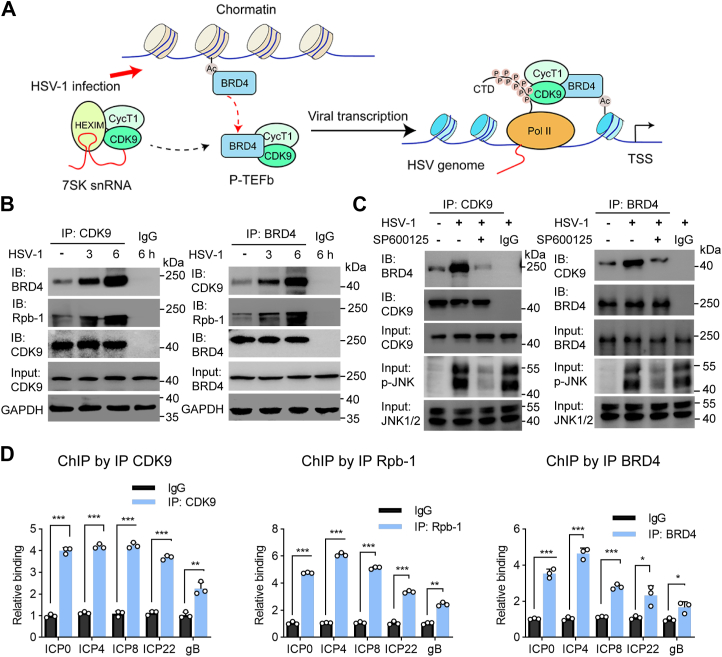
**HSV-1 infection promotes protein complex formation and viral DNA targeting by BRD4-P-TEFb-Pol II.***A*, diagram showing protein complexes in transcription regulation. HSV-1 infection or stress signals trigger BRD4 transition from chromosome association, which in turn recruits P-TEFb to promote gene transcription by RNA Pol II. *B*, HSV-1 infection promotes protein complex formation involving BRD4, CDK9/P-TEFb, and Pol II (Rpb-1 subunit). HeLa cells were infected with HSV-1 (MOI = 0.3) for 3 and 6 h. Protein interaction was assayed by co-immunoprecipitation. Mouse IgG was used as a control for immunoprecipitation. *C*, JNK inhibition blocks protein complex formation by HSV-1 infection. At 6 h post HSV-1 infection, the samples were untreated or treated with 10 μM SP600125 for another hour. The samples were used for protein association by co-immunoprecipitation. Input samples were probed for CDK9, BRD4, total JNK1/2, and phospho-JNK to confirm protein levels and inhibition efficacy. *D*, detection of transcriptional complex to viral DNA using a modified ChIP-qPCR method. HSV-1 infected HeLa cells were formalin-fixed and total DNA was sheared by sonication. The samples were then immunoprecipitated using an antibody as indicated or a mouse IgG as control. The enrichment of viral gene promoter region specific DNA by specific antibody compared to that by corresponding control IgG are presented as mean ± SD (n = 3). ∗, *p* ≤ 0.05, ∗∗, *p* ≤ 0.01, ∗∗∗, *p* ≤ 0.001. The experiments were performed 3 times independently.

As a reader protein, BRD4 uses its bromodomains to associate with acetylated lysine residues for chromatin-targeting. The protein also functions as a transcriptional activator by complexing with proteins such as P-TEFb (20, 33, 34) and RNA Pol II for transcription regulation (23). Next, we questioned whether HSV-1 infection promoted protein association of transcription regulation. To this end, we first performed immunoprecipitation assay and demonstrated BRD4 association with CDK9, a subunit of P-TEFb, and the Rpb-1 subunit of Pol II from HSV-1-infected samples (Fig. 3, *A* and *B*). Importantly, the effect was dependent on JNK activity since SP600125 treatment blocked HSV-1-induced protein complex formation (Fig. 3*C*).

BRD4 recruits P-TEFb and stimulates Pol II-dependent transcription (33, 34, 35). Next, we checked if the proteins were in association with viral DNA. In this regard, we performed a modified version of ChIP assay by immunoprecipitation with P-TEFb (CDK9), Pol II (Rpb-1), BRD4, and a control antibody to detect the associated viral DNA. The presence of viral DNA in the immunocomplexes was quantitatively measured by qPCR. We detected significant enrichment of corresponding viral DNA using promoter region-specific primers for the immediate early genes like ICP0, ICP4, ICP8, ICP22, and late gene gB (Figs. 3*D* and S2). These results indicated that BRD4 released by HSV-1 infection transitioned from chromosome association to viral DNA interaction, potentially for viral gene transcription.

### JNK agonists and chemotherapeutic agents promote BRD4 redistribution and HSV-1 infection

**Figure 4:**
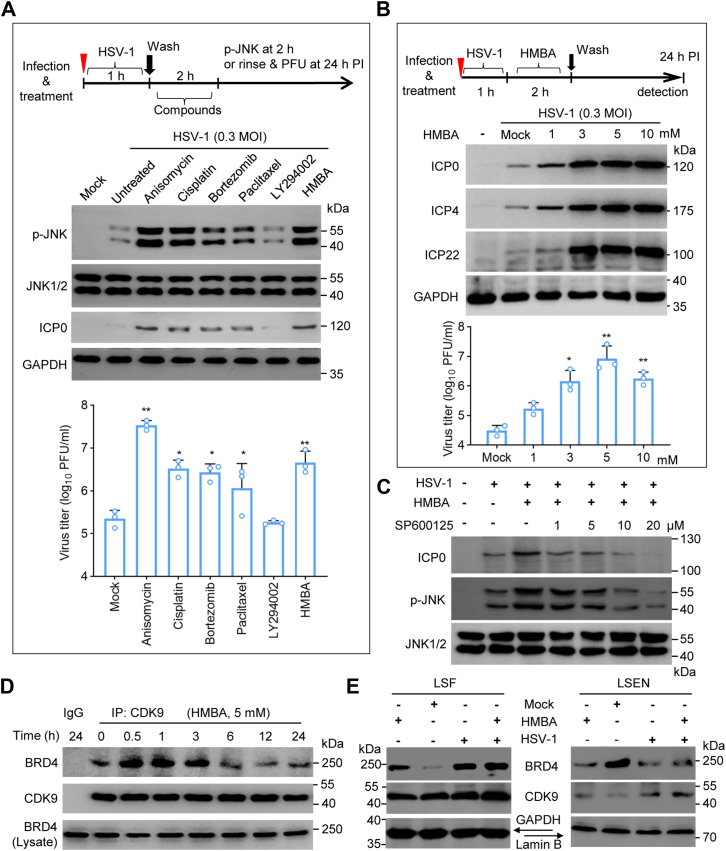
**JNK agonist and chemotherapeutic agents with JNK activation potency on HSV-1 infection.***A*, JNK agonist and antitumor agents on JNK activation and HSV-1 infection. HeLa cells were infected and treated following a scheme as listed. JNK phosphorylation was detected at 2 h post the treatment while ICP0 expression and HSV-1 production was detected at 24 h post infection. *B*, BRD4 release-inducer HMBA on HSV-1 infection. HeLa cells were infected and then treated with HMBA as indicated. The effect of HMBA on HSV-1 infection was determined by detection of HSV-1 protein expression and by a titration assay. *C*, dose effect of HMBA on JNK phosphorylation and HSV-1 infection. HeLa cells were infected with HSV-1 and treated with 3 mM HMBA for 2 h. JNK inhibitor SP600125 was then added at indicated concentrations. JNK phosphorylation and ICP0 expression as an indicator of HSV-1 infection was detected by immunoblotting. *D*, HMBA on CDK9/P-TEFb association with BRD4 determined by co-immunoprecipitation assay. *E*, effect of HMBA on BRD4 release. HeLa cells were infected with HSV-1 or/and treated with 3 mM HMBA for 3 h. The samples were used for BRD4 redistribution assay by fractionation. The experiments were performed 3 times independently. Representative results are presented as mean ± SD. ∗, *p* ≤ 0.05, ∗∗, *p* ≤ 0.01.

To extend our findings on JNK activation and HSV infection, we then investigated whether chemical agents known to activate JNK had the ability to promote HSV-1 infection. To this end, we tested JNK agonists as well as antitumor agents that are known to cause recurrent herpesvirus infection in clinical settings. We also included HMBA as a control for enhancement of HSV-1 infection since the compound has well-documented ability to promote HSV-1 infection (36, 37) and has the ability to release P-TEFb from inactive state to regulate HIV transcription (35, 38). We found several compounds and reagents had the ability to enhance HSV-1 infection as determined by viral protein expression as well as infectious virion production (Fig. 4*A*). We detected increased HSV-1 infection parallelled with increased JNK phosphorylation in the samples treated with chemotherapeutic agents like cisplatin, paclitaxel, and bortezomib, and JNK agonists anisomycin and HMBA (Fig. 4*A*). Their effects were further tested using HMBA since it is a compound with confirmed activity in enhancement of HSV-1 infection (36, 37, 39). Consistent with these reports, we found treatment with HMBA dose dependently enhanced HSV-1 protein expression and HSV-1 infection (Fig. 4*B*). Moreover, we found that the effect was dependent on JNK activity since pretreatment with SP600125 blocked JNK activation and HSV-1 ICP0 protein expression (Fig. 4*C*). Similarly, the effect of bortezomib was also inhibited by SP600125 treatment (Fig. S3). Mechanically, the treatment caused robust interaction between BRD4 and CDK9 within 30 to 60 min after HMBA treatment (Fig. 4*D*). Importantly, the compound also had the ability to induce BRD4 release from chromatin-targeting, since we detected BRD4 redistribution to the LSF fractions of HMBA treated as well as HSV-1 infected samples (Fig. 4*E*). These results thus implied that these chemicals likely promoted HSV-1 infection through induction of BRD4 function transition.

### Animal studies of JNK agonists on HSV-1 infection

**Figure 5:**
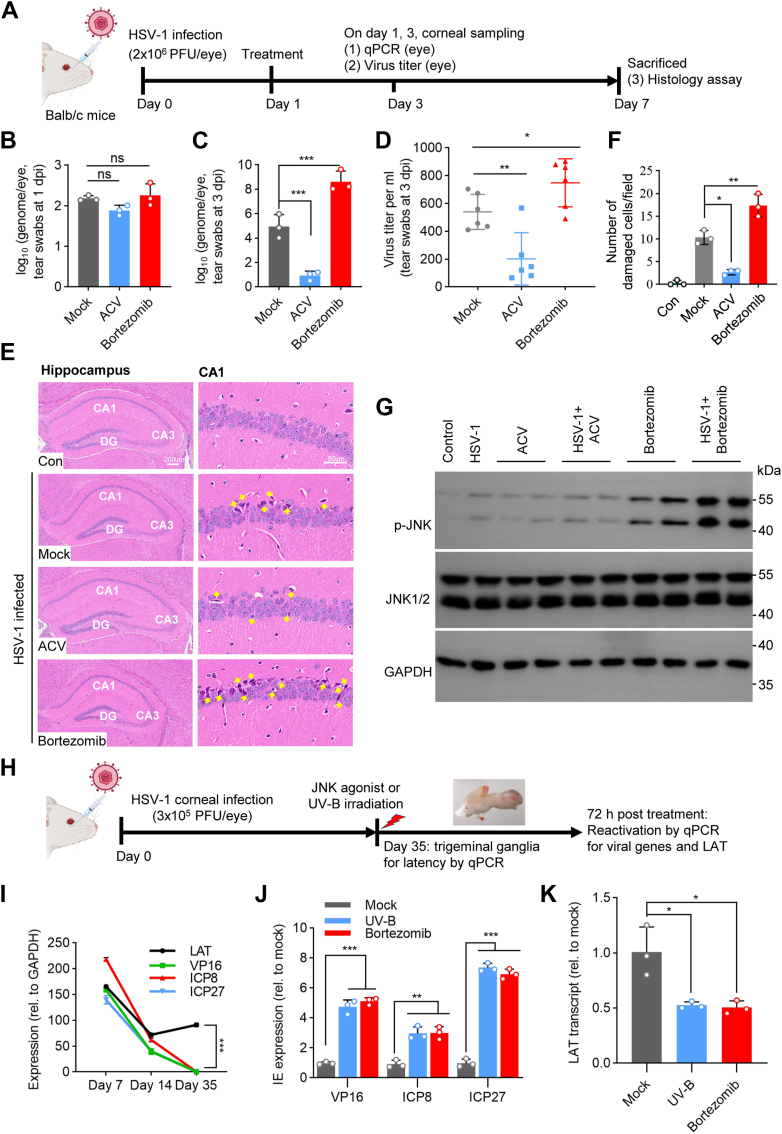
**Effect of bortezomib on JNK activation and on HSV-1 infection in mouse models.***A*, diagram of a mouse model of acute HSV-1 infection. Female Balb/C mice were infected by corneal scarification with 2 × 10^6^ PFU of HSV-1 virus (F strain). *B*–*D*, virus shedding in the tear swabs collected on day 1 (*B*) and day 3 (*C*) and detected by qPCR. A fragment of HSV-1 UL30 DNA was used as a standard for calculation of genome copy number (genome equivalent). *D*, virus titers in tear swabs (n = 6) collected on day 3 determined by plaque forming assay. *E*, representative images of H&E staining of brain tissues from HSV-1 infected mice. The histopathological structure of the hippocampus regions (CA1 and CA3) and the dentate gyrus (DG) (*left panel*) and pathological conditions of the hippocampal region CA1 (*right panel*) were examined after H&E staining. *Arrows* indicate the inflammatory cell infiltration and necrosis of neuronal cells. *F*, numeration of damaged neuronal cells by H&E staining. Data represent mean ± SD of counting from 3 randomly selected fields counted by 2 investigators. *G*, induction of JNK phosphorylation from HSV-1 infected mouse brain tissues. The brain tissues (n = 2) collected from mock-infected or HSV-1 infected mice treated with vehicle or bortezomib at 2 h after the last administration were used for detection of JNK activation by immunoblotting. The antiviral drug acyclovir (50 mg/kg) was included as an antiviral control. GAPDH was used as a loading control. *H*, schematic diagram of bortezomib-induced HSV-1 reactivation. Balb/C female mice were inoculated by the corneal scarification with 3 × 10^5^ PFU of HSV-1. After the establishment of viral latency, the mice were treated with bortezomib (1.5 mg/kg, on day 0 and day 1) or by UVB-irradiation. *I*, the trigeminal ganglia were collected on days as indicated post infection (*I*) and used for detection of LAT transcript or viral gene expression by RT-qPCR. The data are shown as mean ± SD (n = 3). The absence of lytic genes on day 35 shows the establishment of viral latency. *J* and *K*, HSV-1 reactivation in the ganglia determined by RT-qPCR. The ganglia (n = 3) were removed on day 3 and used for detection of viral lytic gene VP16, ICP8, ICP27 (*J*) and LAT (*K*) transcripts. Data are shown as mean ± SD. ∗, *p* ≤ 0.05, ∗∗, *p* ≤ 0.01, ∗∗∗, *p* ≤ 0.001.

To extend these findings, we tested the effect of JNK agonists on HSV-1 infection using a murine herpes simplex keratitis model (HSK). For this reason, we selected bortezomib as it is an anticancer drug clinically linked to recurrent herpesvirus infection (40, 41). Mice were first infected with HSV-1 *via* corneal scarification (Fig. 5*A*). The infected mice were mock treated with a vehicle (0.1% DMSO) or with bortezomib (1.5 mg/kg, ip) at 24 h after inoculation for 3 consecutive days. The antiviral drug acyclovir (ACV) was also included as a control for the study. The presence of HSV-1 in the tears was examined by detection of HSV-1 genomic DNA (Fig. 5, *B* and *C*) as well as of infectious virus (Fig. 5*D*). We detected increased viral DNA and infectious HSV-1 in bortezomib-treated group on day 3 after the infection (Fig. 5, *C* and *D*). The treatment also caused more severe brain inflammation as examined on day 7 after the infection. As shown in Figure 5, *E* and *F*, the brain tissues from mock-treated HSV-1-infected, but not in antiviral drug ACV-treated, group showed signs of nerve cell degeneration, including disintegration and nuclear fragmentation (indicated by arrows). In contrast, aggravated lymphocytic infiltrations and neuronal damage were observed in bortezomib-treated mice (Fig. 5, *E* and *F*), suggesting bortezomib treatment exacerbated HSV-1 disease *in vivo*. The effect seemed to link to JNK activation since intraperitoneal administration of bortezomib induced rapid JNK phosphorylation in HSV-1-infected and bortezomib-treated samples (Fig. 5*G*).

We also used a mouse model of latent HSV-1 infection (Fig. 5*H*) to test the effect of bortezomib on HSV-1 infection (42, 43). After the establishment of virus latency which was confirmed by persistent expression of latency-associated transcripts (LAT), but not genes associated with HSV-1 reactivation in the removed ganglia (Fig. 5*I*), the mice were treated with a vehicle or with bortezomib (1.5 mg/kg) *via* ip injection or by UVB-radiation as a control. Similar to those in UVB-treated group, we detected significant increase of VP16, ICP8 and ICP27 gene expression in the ganglia in bortezomib-treated group (Fig. 5*J*). In addition, we detected reduced presence of LAT (Fig. 5*K*), indicating viral latency rescinding. Thus, chemotherapeutic agents with JNK activation characteristics had the ability to promote HSV-1 infection and *in vivo* reactivation.

Together, these results demonstrated that HSV-1 infection induces JNK1/2 signaling, leading to BRD4 transition from chromatin-targeting to transcription regulation for HSV-1 gene expression.

## Discussion

**Figure 6:**
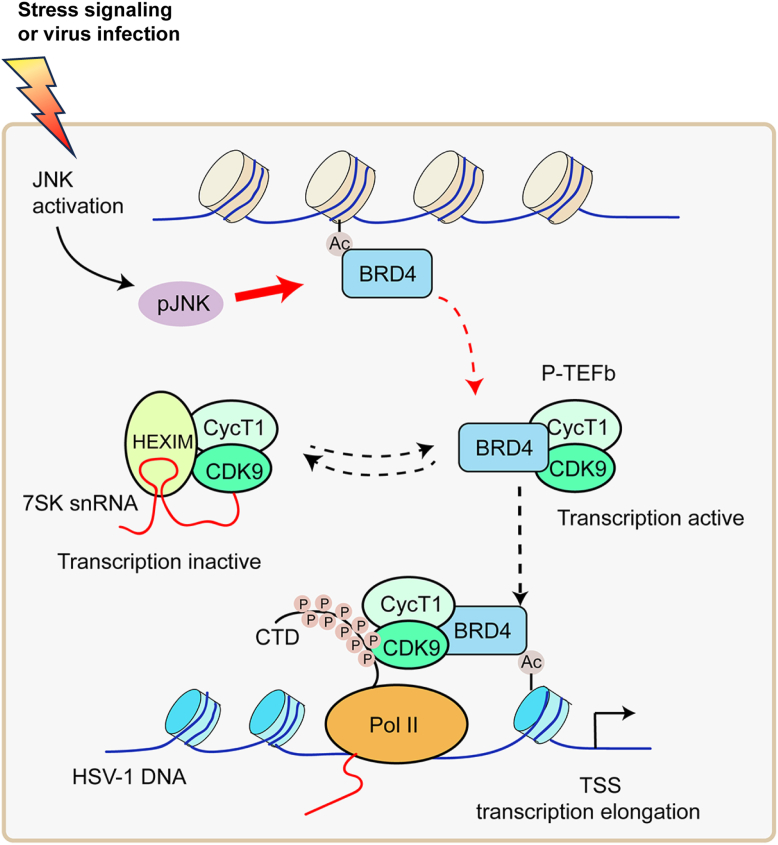
**Diagram of HSV-1 infection and stress signaling on BRD4 function transition.** HSV-1 infection triggers JNK activation, resulting in BRD4 release from chromosome association to gene transcription regulation. Genetic ablation or chemical inhibition of JNK blocks HSV-1 infection by impeding BRD4 release. Chemotherapeutic agents that can induce JNK activation have the ability to promote HSV-1 infection and reactivation through BRD4 function transition.

We show here that HSV-1 productive infection requires JNK pathway signaling. JNK activation by HSV-1 infection or JNK agonist treatment promotes BRD4 release for transcription regulation (Fig. 6). HSV utilizes host transcription machinery, recruiting cellular components or in coordination with viral proteins, to drive the expression of viral gene transcription (44). Infection by HSV-1 causes near-complete loss of Pol II occupancy on the host cell genome (45). The virus relocates Pol II from the cellular genome to its own genome and modulates Pol II activity and transcription, resulting in a coordinated temporal cascade of virus gene expression (46, 47). Previous studies showed that HSV-1 utilizes the viral immediate early genes or tegument proteins that enter the cell as virion components act to manipulate Pol II occupancy on most active host genes (46, 48). RNA Pol II promoter-proximal pausing and release to elongation are key steps regulating HSV-1 transcription (47). The fact that HSV-1 repurposes cellular Pol II for viral transcription within the first 15 min of infection (48) suggests that cellular signaling has a critical role in HSV-1 infection.

The JNK pathway is activated by diverse sets of stimuli, including growth factors, cytokines, proteotoxic, environmental, metabolic stress, and here as we and other groups demonstrated infection. Data presented here along with several reports have demonstrated that BRD4 release requires JNK activity (25, 26, 28). BRD4 is subjected to post-translational modifications (PTMs), including ubiquitination and phosphorylation that regulates the stability of the BRD4 protein and biological function regulation (49). BRD4 harbors multiple conserved consensus sites for phosphorylation by protein kinases (50, 51). We observed little to no BRD4 release from chromatin-targeting in quiescent cells and strong relocation by HSV-1 infection. Unexpected, we found transient expression of a plasmid for BRD4 T1186A/T1212A mutant, a mutant with a critical role in anisomycin and heat shock-induced BRD4 release from chromatin-targeting (28), had little effect against HSV-1 lytic infection (Fig. S1), suggesting other mechanisms may exist. Kristie and colleagues reported JNK-mediated phospho-switch that regulates HSV-1 reactivation and gene expression (16). In addition, other kinases such as CK2 phosphorylates BRD4 to regulate HPV E2-mediated viral transcription (51); while a recent report by Zhang *et al.*, shows that BRD4 phosphorylation at T1186 by the CDK1/cyclin B complex leads to further phosphorylation by PLK1, designating BRD4 for proteasome pathway degradation (52). Thus, further studies are needed to characterize BRD4 modifications in viral infection.

RNA polymerase II promoter-proximal pausing and release to elongation are key steps regulating HSV-1 transcription (47). As a positive regulator of transcription elongation, P-TEFb exists in inactive form by association with the inhibitory complex formed by HEXIM and the 7SK snRNA in quiescent cells and in active state by recruitment and association with Pol II. During gene transcription, Pol II is released from promoter-proximal pausing stage (53). BRD4 activates P-TEFb for Pol II activation and for gene expressions (25, 33, 54). We showed that either HSV-1 infection or chemotherapeutic agents activate JNK and promote BRD4 release from the chromatin. BRD4 persistently associates with chromosomes during mitosis for transmitting epigenetic memory across cell divisions. Stress signals induce the chromatin-bound BRD4 to transit into a transcriptional regulation mode for transcription of signal-inducible genes (25). Thus, virus infection promotes JNK activation for release of BRD4 and its transition to transcription regulation.

Reactivation of viral infections is common in patients undergoing radio- or chemotherapy (40, 55). Previous studies demonstrated that JNK activity is required for HSV replication and reactivation (13, 16). Down regulation of JNK by siRNA or by small molecules like berberine results in inhibition of HSV replication (56). During HSV reactivation, JNK was found on viral gene promoters and regulates *de novo* infection by modulating histone methyl/phosphor switch (16). We show that chemotherapeutic agents such as cisplatin and bortezomib activated JNK pathway and promoted latent HSV-1. Herpetic reactivation and recurrences during radiotherapy is also common. An increased incidence of reactivation of herpesviruses including VZV and HSV-1 has been reported following administration of bortezomib alone or in combinations (57, 58). Active herpes infection can cause interruptions, cessations, or prolongations of treatment and potentially sub-therapeutic care or even deaths due to complications. On the other hand, bortezomib is shown to increase the replication of oncolytic HSV-1 for cancer therapy (59, 60). Thus, understanding JNK activation on HSV-1 replication presents clinical importance.

## Experimental procedures

### Ethics statement

A protocol for the care and use of Balb/C mice in this study was approved by the Institutional Animal Use and Care Committee (IACUC) of Nanjing University (D2103051). All investigations conformed to Jiangsu Province Laboratory Animal License Management Measures and in compliance of the ARVO Statement for the Use of Animals in Ophthalmic and Vision Research (2021 edition).

### Cells and virus

The human cervical carcinoma HeLa cells (ATCC, CCL-2), HEK293T cells (ATCC, CRL-3216), and African green monkey kidney epithelial Vero cells (ATCC, CCL-81) were cultured in DMEM (high glucose) supplemented with 10% heat-inactivated fetal bovine serum (FBS), non-essential amino acids (Life Technologies) and sodium pyruvate at 37 °C in a humidified incubator with 5% CO_2_. The cells were tested routinely for *Mycoplasma* contamination with a PCR detection kit (Beyotime). HSV-1 strain F was propagated in HeLa cells and titrated using Vero cells as previously reported (19).

HeLa cells with JNK1, JNK2 or JNK1 and JNK2 (JNK1/2) knockout were generated in the lab using the CRISPR/Cas9 system. The accession number used for JNK1 and JNK2 was NM_139049 and NM_002752, respectively. A CRISPR design tool (available at crispr.mit.edu) was used to select a single-guide RNA (sgRNA) targeting JNK1 (5′-TCGCTACTACAGAGCACCCG) and JNK2 (5′-GCTGCATTTGATACAGTTCT) and were synthesized by GenScript (Nanjing). To generate ko cells, HeLa cells were transfected with the sgRNA and GenCrispr Cas9-C-NLS Nuclease (GenScript, Z03385) using Lipofectamine 2000 (Life Technologies). Individual clones were selected and validated by DNA sequencing and correctly edited cell clones were confirmed by immunoblotting analysis for the absence of protein expression.

### Reagents and antibodies

Antibodies to JNK1/2 (#554285) was obtained from BD Bioscience, p38 (#9212), Erk2 (#9108), the phosphorylated proteins of MAPK, Rpb-1 CTD (#2629) were purchased from Cell Signaling Technology. Antibodies to CDK9 (Santa Cruz, sc-13130), to BRD4 (Abcam, ab128874), to Lamin B1 (Beyotime, AF5222), to ICP0 (HSV-1 and -2, Santa Cruz, sc-53070), to GAPDH (Bioworld Technology, MB001), to HEXIM1 (CST, #9064), to FLAG (Beyotime, AF519) and HRP-conjugated secondary antibodies (Beyotime) were obtained commercially. Rabbit antiserum to ICP4, ICP22 (HSV-1 and -2) were prepared in the lab using a commercial source (GenScript). The specificity of the antibodies for corresponding proteins was confirmed using cell lysates from transiently transfected or HSV-1 infected cells. The plasmids for mammalian expression of JNK1 (HG10795-UT), JNK2 (HG10745-UT), and BRD4 were purchased from SinoBiological (Beijing), pcDNA5-CMV-FLAG-BRD4 wt was purchased from Miaoling (P29409, Wuhan), and pcDNA5-CMV-FLAG-BRD4-T1186A/T1212A was synthesized by GenScript. Bortezomib (PS-341, S1013), cisplatin (S1166) and BRD4 degrader MZ-1 (S8889) were purchased from Selleck Chemicals (Houston, TX), acyclovir (A3711) from Sigma-Aldrich. Protein A/G agarose beads were purchased from Beyotime. Other chemicals were purchased from Sigma-Aldrich or from Aladdin Scientific (Shanghai).

### Infection and titration assay

For infection assays, cells in culture plates were infected with HSV-1 at MOIs as indicated. The cells were harvested for protein expression or titration studies. For titrations of HSV-1 production, culture supernatants were collected and titrated on Vero cells after series dilutions.

For inhibitory or enhancement effect of a compound on HSV-1 infection, the concentrations of the chemicals were selected based on the reported data (MAPK) or determined in HeLa cells. Since for most of the infection studies the cells were treated with a chemical for 2 h or less, the toxicities of the chemicals like chemotherapeutic agents and HMBA were determined by first treating HeLa cells with a chemical for 8 h followed by further culturing with fresh medium for up to 72 h.

### Immunoprecipitation and immunoblotting studies

Cell lysates were harvested by centrifugation after cell lysis using a buffer containing 50 mM Tris-HCl (pH 7.4), 150 mM NaCl, 1% NP-40, and a cocktail of protease inhibitors (Roche). For immunoprecipitation studies, cell lysates were incubated at 4 °C for 2 h with a capture antibody or a control antibody (IgG), then incubated with protein A/G agarose beads for overnight. The immunocomplexes were collected by centrifugation and washed with ice-cold PBST (0.02% Tween-20 in PBS) three times, and then separated by SDS-PAGE. For immunoblotting, a protein extract (10 μg per lane) was resolved by SDS-PAGE. The protein was then transferred to polyvinylidene difluoride (PVDF) membrane (Millipore, Shanghai), blocked with 5% milk in TBST for 1 h and incubated with a primary antibody at 4 °C for overnight. The signals were detected by incubating with an appropriate HRP-conjugated secondary antibody followed by an ECL reagent kit (Beyotime).

ImageJ was used to measure band intensities (61, 62). Protein levels were normalized to the corresponding loading control. Quantified data were expressed as mean ± SD from at least three independent experiments, the data is provided in Fig. S4.

### Fractionation assay

A modified nuclear fractionation assay was performed to determine the protein distribution and the interactions with chromatin following a reported protocol (25). Briefly, HeLa cells were collected by trypsinization and washed twice with ice-cold PBS. The cells were centrifuged for 5 min (4 °C, 1500×*g*), and the pelleted cell volume (PCV) was recorded. The pellet was resuspended using 5× PCV of ice-cold buffer A (10 mM HEPES pH 7.9, 1.5 mM MgCl_2_, 10 mM KCl, 1 mM DTT, 0.5 mM PMSF, and a protease inhibitor cocktail) and left on ice for 10 min in order to swell cells. After centrifugation at 1500×*g* to pellet cells, the swollen cells were then subjected to low-salt extraction by re-suspending in 2× PCV of buffer A plus 1% NP-40 for 10 min to break the cell membrane. The supernatant was collected after centrifugation (5000×*g*, 4 °C, 5 min) and the volume recorded (about 3.6× PCV). After rinse with 5× PCV of buffer A, the nuclei pellet was further extracted 3 times with 1× PCV of buffer A plus 0.5% NP-40 and 75 mM NaCl for 5 min with occasional vortex. The supernatant, named low salt fraction (LSF) was collected by centrifugation. The volume of the pooled LSF supernatants from three extractions was about 5.6× PCV. Subsequently, the remnant of low salt extracted nuclei (LSEN) was resuspended to 5.6× PCV of 1× Laemmli sample buffer (with 1% SDS). The LSF and LSEN fractions were then subjected to immunoblotting analysis.

### Transfection and RNA interference experiments

For transfection studies, HeLa JNK1/2 ko cells were transfected with plasmids encoding human JNK1 (HG10795-UT), JNK2 (HG10745-UT), or a combination of both plasmids using Lipofectamine 2000 according to the manufacturer’s instructions. The cells were used at 48 h after transfection for infection and for protein expression assays.

To suppression JNK expression, HeLa cells were transfected using Lipofectamine 2000 according to the manufacturer’s instructions. Specific siRNAs targeting JNK1: GACCAUUUCAGAAUCAGACUU, JNK2: GAUGCUAACU UAUGUCAGGUU, and a non-targeting scrambled control siRNA (UUCUCCGAACGUGUCACGUTT) were designed according to the cDNA sequence of JNK1/2 and were purchased from GenePharma (Shanghai).

### RT-PCR and real-time PCR assay

Genomic DNA and total RNA from all samples were extracted using a MiniBEST viral RNA/DNA extraction kit (TaKaRa, 9766) or with TRIzol reagent (Life Technologies) to extract RNA. cDNA was synthesized using the HiScript RT Super Mix (Vazyme, R323-01) according to the manufacturer’s instructions. The RT Primer Mix contains oligo (dT) and random primer. The LAT was transcribed with a reverse primer. Real-time PCR was performed using SYBR Green PCR Master Mix (Q141-02/03, Vazyme, Nanjing) on an ABI 7300 real-time PCR system (Applied Biosystems). The primers are listed in Table 1. Data was analyzed using the 2^-△△Ct^ method to obtain relative abundance (63). The GAPDH Ct level was used as an internal control for value normalization. Any product detected above 32 cycles was deemed unreliable and thus considered negative.

**Table 1:** List of primer pairs used for PCR and qPCR studies^a^

Gene	PCR primers	Product size
ICP8	5′-GGAGGTGCACCGCATACC 5′-GGCTAAAATCCGGCATGAAC	56 bp
ICP27	5′-TCATGCACGACCCCTTTGG 5′-CTTGGCCCGCCAACAC	75 bp
LAT	5′-GCATAGAGAGCCAGGCACAAAA 5′-ACGTACTCCAAGAAGGCATGTG	61 bp
VP16	5′-TCGGCGTGGAAGAAAC 5′-GAGACGAACGCACCCAAAT	83 bp
UL30	5′-CGCGCTTGGCGGGTATTAACAT 5′-TGGGTGTCCGGCAGAATAAAGC	115 bp
GAPDH	5′-ACAGTCAGCCGCATCTTCTT 5′-ACGACCAAATCCGTTGACTC	97 bp

a The oligo sequences of HSV-1 were based on HSV-1 strain F (GenBank accession number GU734771), and NM_002046 was used for *GAPDH*.

### Chromatin immunoprecipitation (ChIP)

A modified chromatin immunoprecipitation assay was carried out to determine host protein interactions with viral DNA as previously described (19, 64). The immunoprecipitated protein-DNA complexes were incubated at 65 °C for overnight to reverse the cross-linking and with proteinase K at 55 °C for 1 h to remove proteins prior to immunoprecipitation. The immunoprecipitated DNA was purified by phenol chloroform extraction, followed by ethanol precipitation. To quantitatively measure DNA levels in the immunocomplexes, qPCR was performed using those in the IgG controls for comparison. The primers for viral DNA detection are listed in Table S1.

### Animal studies

Inbred female BALB/C mice were purchased from Beijing Vital River Laboratory Animal Technology Co., Ltd. For ocular HSV-1 infection, mice were anesthetized by IP injection of ketamine (100 mg/kg) and xylazine (5 mg/kg). Following corneal scarification of the right eye with a 28-gauge needle, 2 × 10^6^ PFU of HSV-1 was applied to infect the mice. For drug treatment, the mice were injected with a vehicle (0.1% DMSO, 100 μl *via* ip route) or with bortezomib (1.5 mg/kg, 24 h after inoculation and for 3 consecutive days). Daily treatment with acyclovir (50 mg/kg) was included as a control for antiviral treatment. For protein phosphorylation assay, mice (n = 2) were killed at 2 h after the last injection of bortezomib and the brain tissues were used for immunoblotting studies. For detection of virus, the animals were monitored every 24 h, and ocular swabs were collected on day 1 and day 3 following the treatment. The animals were killed on day 7 post infection and brain tissues (n = 3) were collected and fixed in 4% paraformaldehyde for detection of brain tissue damage (65) with hematoxylin-eosin (H&E) staining. The images were captured with an Olympus VS200 slide scanner.

A mouse model of latent infection was used to test HSV-1 reactivation (43, 66). The latency was confirmed by detection of the LAT and viral lytic genes in the ganglia. After the establishment of viral latency (counted as day 0, about 5 weeks after the initial inoculation), the uninfected or HSV-1 latently infected-mice were injected *via* the ip route with a vehicle (0.1% DMSO) or with bortezomib (1.5 mg/kg, on day 0 and day 1). A group by UVB irradiation was included as a positive control for HSV-1 reactivation by exposing the eyes of latently infected mice to 250 mJ/cm^2^ of UVB light following a reported protocol (67). Mice were monitored every 24 h. On the third day post the first treatment, the mice were euthanized, and the trigeminal ganglia (TG) were collected for detection of lytic gene and LAT expression by RT-qPCR.

### Statistical analysis

Statistical analysis was performed using SPSS 17.0 software package. For comparisons between two groups, an unpaired two-tailed Student’s *t* test was used and one-way ANOVA for multiple group comparisons. Data are presented as mean ± standard deviation (SD), using ∗ *p* ≤ 0.05, ∗∗*p* ≤ 0.01, and ∗∗∗*p* ≤ 0.001 to show statistical significance and ns for no significance.

## Data availability

Data used during the current study are available from the corresponding author on reasonable request.

## Supporting information

This article contains supporting information (64).

## Conflict of interest

The authors declare that they have no conflicts of interest with the contents of this article.
