# Supplementary material for: Activation of the stress-activated protein kinase JNK in response to herpes simplex virus-1 infection coordinates transition of BRD4 from chromosome association to transcription elongation
Source: J Biol Chem. 2025 Aug 12;301(9):110590. doi: 10.1016/j.jbc.2025.110590 (PMC12450640; doi:10.1016/j.jbc.2025.110590)
Supplement: Supporting Figures and Tables [file mmc1.docx]

**Supporting Information**

**Activation of the stress-activated protein kinase JNK in response to herpes simplex virus-1 infection coordinates transition of BRD4 from chromosome association to transcription elongation**

Weikang Sun^1,2,#^, Ke Ren^3,#^, Jingjing Li^2,#,%^, Mengyu Zhang^1,2^, Qishen Jiang^1,4^, Lingling Wang^5,*^, Erguang Li^1,2,6,*^

^1^ State Key Laboratory of Pharmaceutical Biotechnology and Medical School, Nanjing University, 22 Hankou Road, Nanjing, Jiangsu, 210093, China

^2^ Jiangsu Key Laboratory of Molecular Medicine, Medical School, Nanjing University

^3^ School of Laboratory Medicine, Chengdu Medical College, Chengdu, China

^4^ College of Life Sciences, Nanjing University, Nanjing, China

^5^ School of Medical Informatics, Xihua University, Chengdu, Sichuan, China

^6^ Institute of Medical Virology, The Affiliated Drum Tower Hospital of Nanjing University Medical School, Nanjing, China

^%^ Current address, Zhejiang Provincial Hospital, Hangzhou, Zhejiang, China

# contributed equally,

***** For correspondence,

Erguang Li, PhD, at erguang@nju.edu.cn, ORCID ID: 0000-0002-3065-1336

**Lingling Wang, PhD, at wanglingling@mail.xhu.edu.cn, ORCID ID: 0000-0002-2556-4692**

**Content and page number**

**Table S1…………………..…………………………………………..2**

**Figure S1****…………………..…………………………………………..3**

**Figure S2…………………..……………………………………….….4**

**Figure S3…………………..……………………………………….….5**

**Figure S4…………………..………………………………………..6-7**

**Table S1. Primers used for gene-specific detection of viral DNA in immunocomplexes***

| **Gene** | **Primers for ChIP analysis** | **Location** | **Production size** |
| --- | --- | --- | --- |
| ICP4^PR^ | 5’-CTATATGAGCCCGAGGACGC  5’-CGTCTGACGGTCTGTCTCTG | -31 to +68 bp | 100 bp |
| ICP4^UNPR^ | 5’- CGCGTACGAACACGTCGAT  5’- TTTATCACCACCATGGCCC | -2000 bp | 110 bp |
| ICP4^DNPR^ | 5’- GGAGTTTCTGGGGCTGCTC  5’- GTCGCACGCCAGGTAGG | +1300 bp | 127 bp |
| gB^PR^ | 5’-TGGGTGGAGTGATCAAAGAG  5’- GCATCACCCATCGCTTCT | -257 to -201 bp | 57 bp |
| GAPDH^PR^ | 5’-TTCGACAGTCAGCCGCATCTTCTT  5’-CAGGCGCCCAATACGACCAAATC | +44 to +153 bp | 110 bp |
| ICP0^PR^ | 5’-ATAAGTTAGCCCTGGCCCCGA  5’-GCTGCGTCTCGCTCCG | -29 to +35 bp | 65 bp |
| ICP8^PR^ | 5’-CCACGCCCACCGGCTGATGAC  5’-TGCTTACGGTCAGGTGCTCCG | -107 to +20 bp | 128 bp |
| ICP22^PR^ | 5’-TATTAGGGCGAAGTGCGAGC  5’-CTTTATGTGCGCCGGAGAGA | -28 to +72 bp | 101 bp |

***** The oligos used for detection of HSV-1 promoter region DNA were based on HSV-1 strain F (accession number GU734771). The locations refer to regions relative to the predicted transcription start site (TSS) of corresponding genes. PR stands for promoter region, while UNPR and DNPR represent the non-promoter regions upstream or downstream relative to the promoter region.

**
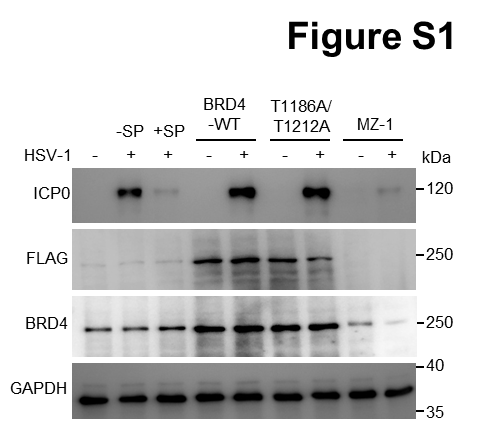
**

**Figure S1.** **Effect of transient expression of BRD4 T1186A/T1212A mutant on HSV-1 lytic infection.**

293T cells were transiently transfected with a plasmid expressing wild-type (WT) BRD4 or BRD4 T1186A/T1212A for 48 h, followed by HSV-1 infection (MOI=0.3, 24 h). BRD4 expression and HSV-1 ICP0 production were detected by immunoblotting. As a control for the assay, PROTAC BRD4 degrader MZ-1 (0.3 μM) was included to demonstrate the requirement of BRD4 for HSV-1 infection. The requirement of JNK was confirmed by JNK inhibition with SP600129 (10 μM). GAPDH was used as loading control. The experiments were performed 3 times independently.


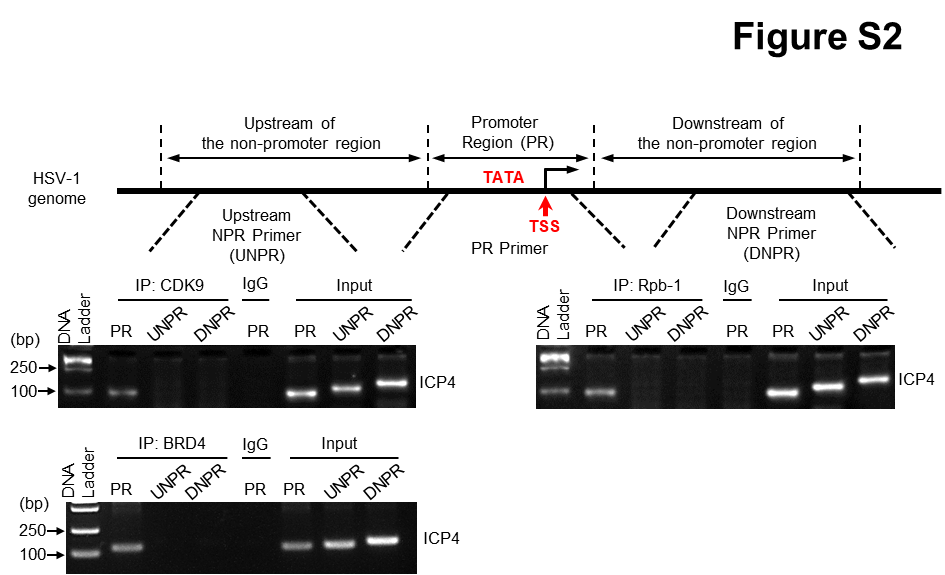


**Figure S2. Validation of primer specificity by PCR.**

HSV-1 infected or uninfected control cells were formalin-fixed following a protocol for ChIP assay (Dahl, J.A. and Collas, P. *Nature Protocols*, 3(6):1032-1045 (2008). The total DNA from the samples was sheared by sonication. The samples were immunoprecipitated with antibody against CDK9/P-TEFb, Rpb-1/Pol II, or BRD4. The presence of viral DNA was detected by PCR using ICP4 as an example. PR stands for primers targeting the promoter region, while UNPR and DNPR represent the non-promoter regions upstream or downstream relative to the promoter region (Table S1).

**
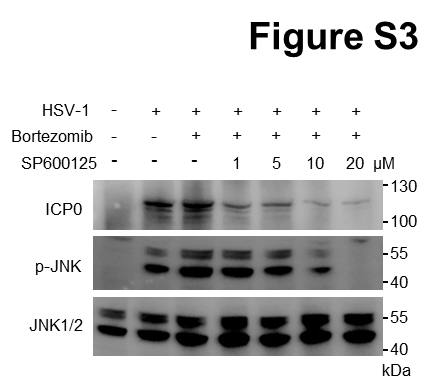
**

**Figure S3. Effect of bortezomib on JNK phosphorylation and on HSV-1 infection.**

HeLa cells were infected and then treated with 10 μM bortezomib in the absence or presence of varying amount of SP600125 for 2 h. JNK phosphorylation and ICP0 expression were detected by immunoblotting.

**
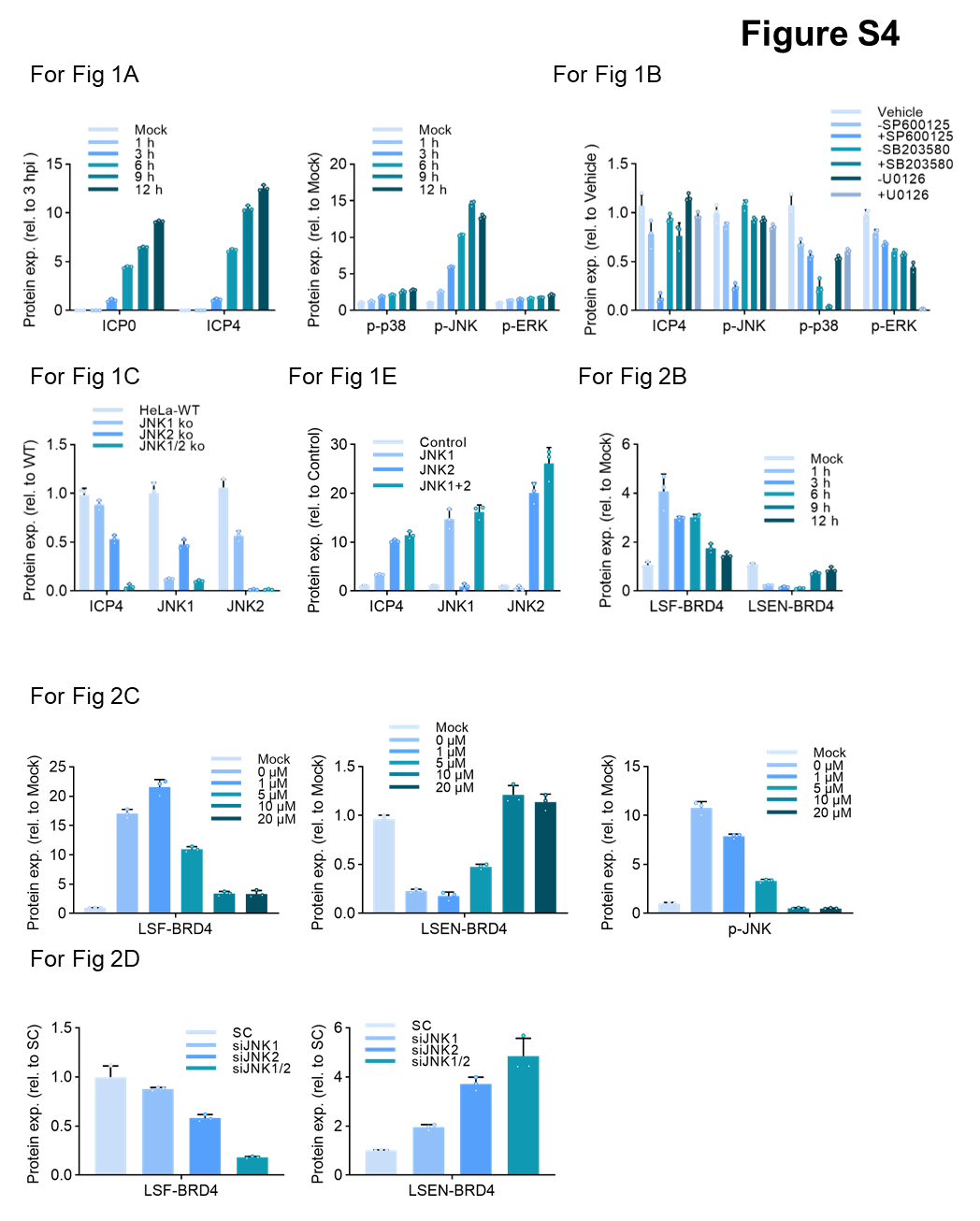
**

**
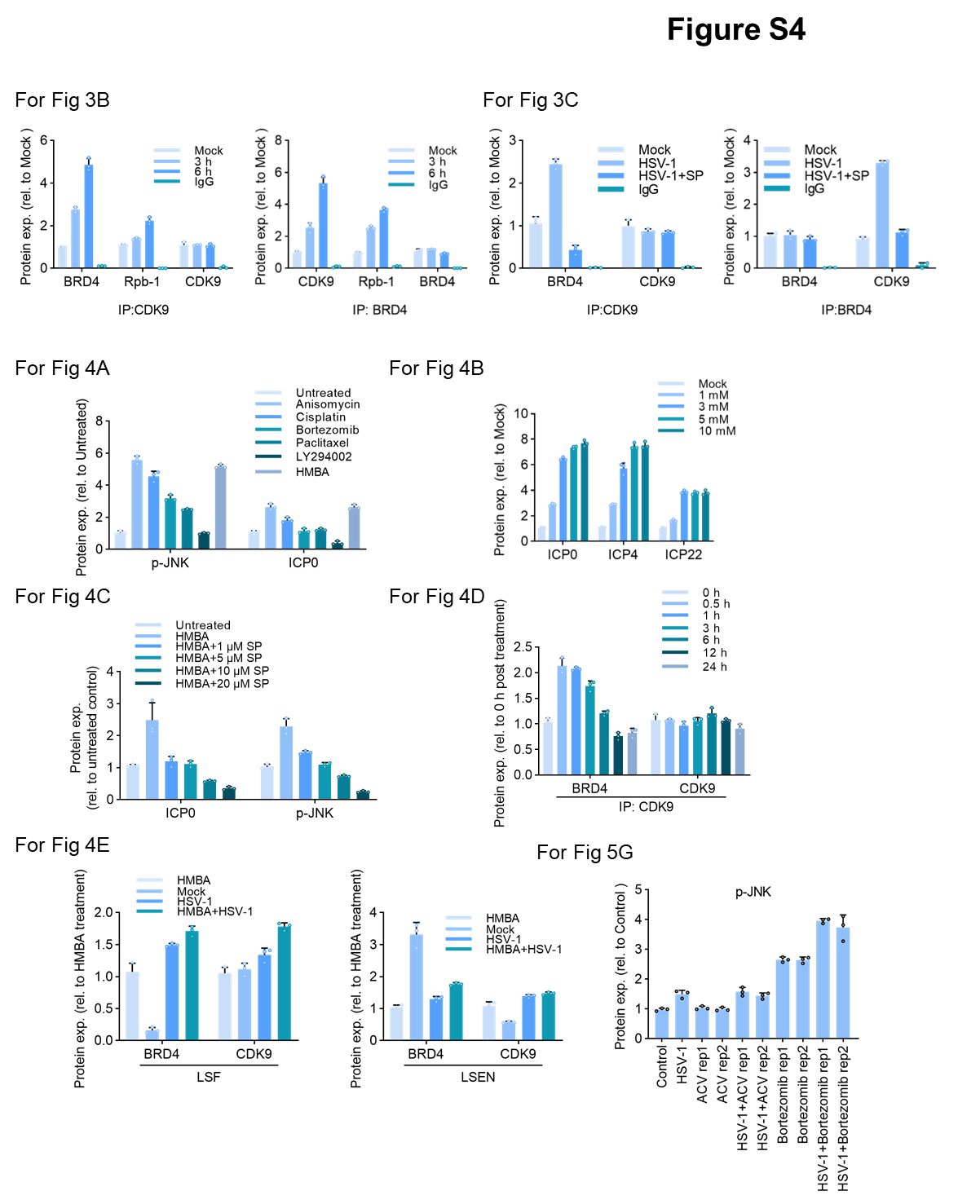
**

**Figure S4. Quantitation of immunoblotting analysis in this study.**

The gel bands presented in the corresponding figures were quantified using ImageJ and presented as mean ± SD.
